# Pleurobiliary fistula, a rare complication of hepatocellular carcinoma after locoregional chemotherapy: a case report

**DOI:** 10.4076/1757-1626-2-7992

**Published:** 2009-06-19

**Authors:** Spiros G Delis, Dimitrios Karakaxas, Andreas Bakoyiannis, Konstantina Paraskeva, Christos Dervenis

**Affiliations:** 1 Liver Surgical Unit, Agia Olga HospitalAgias Olgas 3-5, 142 33, AthensGreece; 2 Department of Gastroenterology, Agia Olga HospitalAgias Olgas 3-5 142 33, AthensGreece

## Abstract

A rare complication of the compilation of high intrahepatic biliary pressure and the formation of a subdiaphragmatic abscess is that of pleurobiliary fistula. We present a case of 67-year-old male who presented with pleurobiliary fistula following transarterial chemoembolization in a patient with a large hepatocellular carcinoma, as well as the course of the diagnostic procedures and the therapeutics interventions which took place.

## Introduction

Pleurobiliary fistula is a rare event related to high intrahepatic biliary pressure [1] and subdiaphragmatic abscess formation ruptured in the pleural space. It may present as a complication of trauma [2], hydatid disease [3] or pyogenic abscess. Four cases of hepatocellular carcinoma (HCC) have been reported as a cause of bronchobiliary fistula in the surgical literature. Three of them underwent palliative measures and eventually died from their disease. A single case of surgical resection of both liver tumor and involved right lower lung lobe demonstrated a better outcome. Herein we present a case of pleurobiliary fistula created as a complication of transarterial chemoembolization (TACE) in a patient with large HCC treated by both endoscopy and pleurodesis.

## Case presentation

A 67-year-old Caucasian male presented with hepatitis B cirrhosis and a central tumor lesion in the liver. Radiologic imaging confirmed the diagnosis of HCC. The tumor, measuring 12 cm in diameter invaded both right and left liver lobe. Selective TACE was performed of the tumor feeding vessels from the right hepatic artery. TACE, performed by injection of lipiodol and epirubicin emulsion followed by cyano-acrylate particles, was successful.

**Figure 1.**
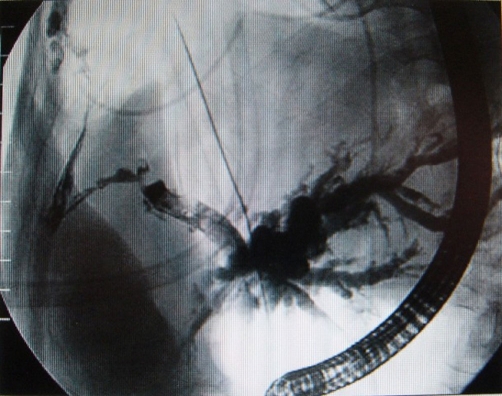
Image of endoscopic cholangiography showing the route of the pleurobiliary fistula as well as the distension of the intrahepatic biliary tree.

TACE was repeated twice in a 6 month period but after the last session the patient was admitted in the Liver Surgical Unit of “Agia Olga” Hospital with fever and shortness of breath. Computed tomography (CT) of the abdomen revealed a necrotic liver mass with left lobe biliary dilatation associated with bile duct invasion from the tumor. Chest X- ray and CT scan demonstrated significant right pleural effusion and localized pneumonia. Blood test exhibited hypoalbuminemia and mild liver dysfunction with increase of the cholestatic enzymes. A decision for chest-tube drainage of the pleural effusion was made due to hypoxia and high grade fever (T > 38.5 C). Two liters of bilious fluid were drained initially followed by 500 cc of daily drainage. Pleural fluid examination revealed high bilirubin level (17 mg/dl) associated with pleuro-biliary fistula. Endoscopic retrograde cholangiography (ERC) (Figure 1) confirmed the presence of a partially obstructed common bile duct with pleuro-biliary communication. A 12 French Silastic stent was placed to decompress the intrahepatic biliary system and intravenous hyperalimentation was induced. However, chest tube drainage remains required pleurodesis. Despite successful management of pleural drainage the patient succumb from sepsis and liver failure 15 days later.

## Discussion

Pleurobiliary fistula is a rare event usually presented in patients with liver hydatid disease. Hydatid cyst may rupture in the pleural space after invasion of the diaphragm. Two major factors are required such as the mechanical effect of an increased intraluminal pressure of the biliary system due to bile duct obstruction and the local inflammatory process triggering adhesion between the liver and the diaphragm. Bronchobiliary fistula was reported also in patients with large HCCs when they underwent TACE. In the present case invasion of the common bile duct from the HCC resulted in common bile duct obstruction with subsequent increase of the intrabiliary pressure. The formation of the subphrenic liver abscess was attributed to TACE. These predisposing factors led to rupture of the abscess in the pleural space. Invasion of the bile ducts due to HCC is a rare clinical picture occurred in 2% of all HCC cases. Intrabiliary tumor growth is related with necrosis and bile duct obstruction from devitalized fragments. This devastating situation correlates with early death as reported by Kojiro and associates.

A diagnosis of bronchobiliary and pleurobiliary fistula is based on radiologic imaging findings. CT scan is non-specific to identify the biliopleural communication. Cholangiography and Tc-HIDA scan are the techniques of choice to establish the diagnosis.

Treatment of broncho- and pleurobiliary fistula is surgical [4] although few patients tolerate surgical intervention, but it can also be treated with conservative measures [5]. Tumor resection along with the fistulous tract is recommended. Segmental lung resection is also advised in cases of bronchobiliary fistula as recently reported. Malnutrition and sepsis restrict the efficacy of such a demanding operation and a recent shift to non-surgical interventions has been described in the literature by endoscopic stent placement [5]. Palliative methods offered a short term improvement followed by fistula recurrence due to failure of these methods to address the mechanism of pleurobiliary fistula formation.

Death is almost inevitable in these cases because of respiratory failure or disease progression.

In our case, chest tube drainage of the pleural effusion documented the diagnosis of pleurobiliary fistula, which was further confirmed by ERC. Due to persistence of chest tube drainage, a stent was placed initially to decompress the biliary system followed by pleurodesis.

Because of the poor liver function and sepsis, the patient eventually died; although pleural drainage was decreased with the previous mentioned conservative measures.

## List of abbreviations

CT: Computed tomography
ERC: Endoscopic retrograde cholangiography
HCC: Hepatocellular carcinoma
TACE: Transarterial chemoembolization
